# A monolithically integrated in-textile wristband for wireless epidermal biosensing

**DOI:** 10.1126/sciadv.adj2763

**Published:** 2023-11-10

**Authors:** Xiaohao Ma, Pengwei Wang, Liting Huang, Ruochen Ding, Kemeng Zhou, Yuqing Shi, Fan Chen, Qiuna Zhuang, Qiyao Huang, Yuanjing Lin, Zijian Zheng

**Affiliations:** ^1^ School of Microelectronics, Southern University of Science and Technology, Shenzhen 518055, China.; ^2^ Laboratory for Advanced Interfacial Materials and Devices, School of Fashion and Textiles, The Hong Kong Polytechnic University, Kowloon, Hong Kong SAR 99077, China.; ^3^ Research Institute for Intelligent Wearable Systems, The Hong Kong Polytechnic University, Kowloon, Hong Kong SAR 99077, China.; ^4^ Department of Applied Biology and Chemical Technology, Faculty of Science, The Hong Kong Polytechnic University, Kowloon, Hong Kong SAR 99077, China.; ^5^ Research Institute for Smart Energy, The Hong Kong Polytechnic University, Kowloon, Hong Kong SAR 99077, China.

## Abstract

Textile bioelectronics that allow comfortable epidermal contact hold great promise in noninvasive biosensing. However, their applications are limited mainly because of the large intrinsic electrical resistance and low compatibility for electronics integration. We report an integrated wristband that consists of multifunctional modules in a single piece of textile to realize wireless epidermal biosensing. The in-textile metallic patterning and reliable interconnect encapsulation contribute to the excellent electrical conductivity, mechanical robustness, and waterproofness that are competitive with conventional flexible devices. Moreover, the well-maintained porous textile architectures deliver air permeability of 79 mm s^−1^ and moisture permeability of 270 g m^−2^ day^−1^, which are more than one order of magnitude higher than medical tapes, thus ensuring superior wearing comfort. The integrated in-textile wristband performed continuous sweat potassium monitoring in the range of 0.3 to 40 mM with long-term stability, demonstrating its great potential for wearable fitness monitoring and point-of-care testing.

## Teaser

The monolithically integrated in-textile biosensing system sheds light on the advances of textile-based wearable electronics.

## INTRODUCTION

Intensive research efforts in wearable bioelectronics have rapidly expanded their practical applications in personalized health monitoring (*1*–*11*). Wireless and wearable devices offer a noninvasive way of extracting physiological parameters related to health status and transmitting data to a user device in a real-time and continuous manner (*12*–*15*). Sweat as an attractive biofluid containing numerous molecular biomarkers, including electrolytes, metabolites, amino acids, and hormones, can be analyzed using a variety of bioelectronics (*16*). Continuous monitoring of these biomarkers enables real-time monitoring of daily health status and early disease detection and management (*17*–*23*).

With the evolution of materials and electronics, textiles have become one of the most popular materials for wearable bioelectronics because of their superiorities including permeability, biocompatibility, and comfortable skin contact for efficient epidermal biosensing. Prolific research efforts have been devoted to applying electronic textiles (E-textiles) for noninvasive sensing of critical biomarkers such as sweat sodium, potassium, ammonium, pH, glucose, and lactate (*24*–*30*). For example, functional textile systems can be fabricated by coating materials such as conductive fillers into a porous textile structure via screening printing (*31*–*34*). Nevertheless, these approaches are prone to failures such as cracks and delamination due to mechanical mismatch. Alternatively, button-like electronics consisting of sensors, batteries, and wireless communication components that can be directly attached to existing clothing have been developed (*35*, *36*), whereas the fraying of conductive threads on the stitching could eventually cause a short circuit between neighboring stitches. Digital embroidery as another popular embedded form for E-textiles has both high electrical conductance and mechanical robustness without compromising the durability or breathability of the underlying clothing (*37*–*39*). However, the commonly adopted electronic package such as small outline integrated circuit requires pin interspace of less than 1.77 mm (0.05 inch) (*40*). These approaches pose limitations on the reliable functionality of mechanical interference and system miniaturization, mainly because of the discrete interconnects between electronic components, especially when applied to chips with dense pins.

Therefore, in-textile fabrication and monolithic integration of electronic devices and circuits using commonly adopted conductive materials (e.g., tin and copper) are in high demand (*41*–*44*). Polymer-assisted metal deposition (PAMD), a low-cost and high-throughput technique, has become a practical chemical process for fabricating conductive textiles due to its superior stability and conductivity (*45*–*50*). For instance, in-textile metallic Cu cloth fabricated with PAMD showed superior conductivity up to 2.0 × 10^7^ S m^−1^, which is typically in the same order of magnitude as the bulk Cu metal (6.41× 10^7^ S m^−1^) (*51*). In addition, conductive cloth enabled by PAMD has an interpenetrative network of polymer/metal forms at the interface, which significantly improves the adhesion between the metal and the substrate (*52*). So far, the remarkable flexibility and mechanical stability of conductive patterns fabricated by PAMD have not yet been fully leveraged to achieve monolithic sensing systems.

Here, we report a monolithically integrated in-textile wristband for real-time wireless sweat potassium ion (K^+^) analysis by PAMD. The as-fabricated textile electrochemical sensing system enables electrochemical signal processing and transmission by creating a monolithic in-textile circuit based on a permeable metallic cloth with high durability against bending, folding, and crumpling. In addition, double-sided photolithography modified for in-textile patterning is adopted to achieve circuit patterns on the monolithic cloth. Its compatibility with PAMD allows the precise reproduction of well-designed patterns by imitating the traditional printed circuit board manufacturing process. Moreover, such an in-textile metallic patterning strategy enables an excellent air permeability of 79 mm s^−1^ and moisture permeability of 270 g m^−2^ day^−1^. These are competitive compared with commercial textile fabrics and show more than one order of magnitude higher than commercial medical tapes and elastomers that are commonly adopted in flexible electronics. Therefore, the comfort of such wearable bioelectronics can be largely improved. The as-fabricated in-textile sensor can detect K^+^ in the sweat with a high sensitivity of 66 mV/decade, good selectivity, and long-term stability. As a proof of concept, K^+^ concentrations in volunteer perspiration are monitored using the textile system, and the standard tests performed by the high-performance inductively coupled plasma mass spectrometry (ICP-MS) verify the results from the textile wristband. The system can perform electrochemical detection with a high goodness of fit of 0.994 and realize accurate biosignal transmission to the mobile. This work provides a promising strategy for fabricating integrated and wearable bioanalysis devices, demonstrating its potential applications in fitness monitoring, digital health, and the Internet of Medical Things.

## RESULTS

### Design and fabrication of the monolithically integrated in-textile wristband

**Fig. 1.**
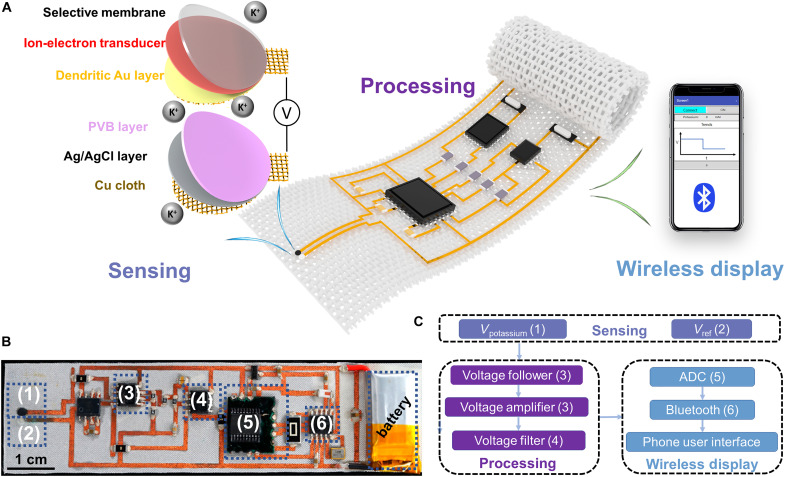
Image and schematic illustration of the monolithically integrated in-textile wristband. (**A**) Schematic of textile wristband for wireless sweat K^+^ analysis. (**B**) Photograph and schematic illustration of the flexible patch (2.5 cm by 9 cm). (**C**) The logical flow of the systematic design. Components 1 and 2 are the working electrode and reference electrode, respectively, for electrochemical sweat sensing. Components 3 and 4 are the voltage follower, amplifier, and filter for data processing. Components 5 and 6 are the analog-to-digital converter (ADC) and Bluetooth module, respectively, for wireless signal display.

The monolithically integrated in-textile wristband was composed of the following components: a highly selective sensor for K^+^ detection, a voltage conditioning circuit for signal extraction and processing, and a Bluetooth module for wireless data transmission and mobile visualization (Fig. 1A). The K^+^ ion-selective sensor was based on a two-electrode configuration consisting of the ion-selective electrode as the working electrode and the Ag/AgCl solid-state reference electrode with a diameter of 1.5 mm. The measurement of K^+^ levels was facilitated through the ion-selective electrodes coupled with a polyvinyl butyral (PVB)–coated reference electrode to maintain a stable voltage in solutions at different ionic strengths. By drop-casting poly(3,4-ethylenedioxythiophene) polystyrene sulfonate (PEDOT:PSS) as an ion-to-electron transducer on a working electrode, the robust potentiometric sensor was obtained for continuous measurements with negligible voltage drift. Last, Ecoflex was applied as insulating glue to the area of the electrical routing design to improve the circuit stability and maintain the permeability across the textile substrate. The biosignal extraction was based on an open-circuit potential (OCP) method, and a high-impendence potential detection circuit was designed to realize the signal sensing, processing, and wireless transmission while eliminating interference signals (Fig. 1B and fig. S1A). Note that the in-textile circuit design was based on a one-layer configuration to maintain the breathable fabric structure while ensuring the conductivity of the in-textile interconnects (fig. S1B). Figure 1C displays the schematic diagram of the in-textile circuit design and illustrates the system flow chart for real-time and wireless sweat analysis. After the analog signal was obtained by the electrochemical sensor, the voltage follower eliminated the high impedance of the signal. With proper impendence matching for signal amplification and filtering modules, the output signal was finely resolved within the range of the analog-to-digital converter. The microcontroller capable of computational and serial communication functions was used to calibrate, compensate, and transmit the extracted signals to an onboard wireless transceiver. The transceiver facilitated wireless data transmission to a Bluetooth-enabled mobile handset with a custom-developed application. Subsequently, the application displayed the received data strings in graphic form in a real-time manner and performed automatic data downloads so as to enable a user-friendly experience.

**Fig. 2.**
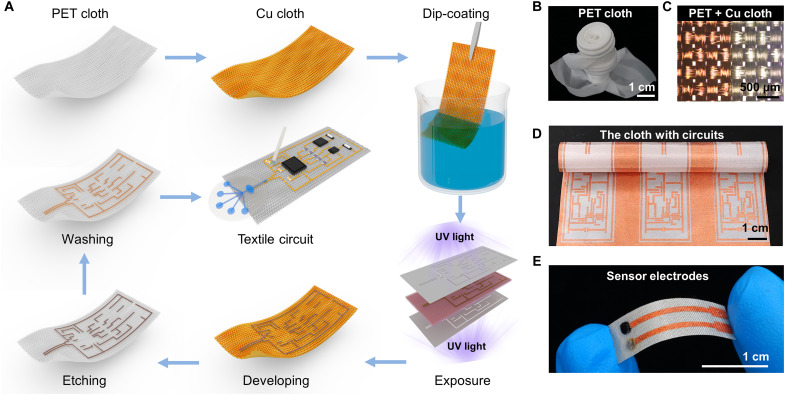
Manufacturing process of the textile wristband and related photographs. (**A**) The fabrication process of double-sided photolithography for conductive interconnects and system assembly. (**B**) Versatile folded with PET cloth, indicating its excellent flexibility. (**C**) The pattern edge of Cu interconnects, showing the sharp edges of patterned metallic textiles enabled by the double-sided photolithography. (**D**) A large-size PET cloth patterned with complicated Cu circuits. (**E**) A flexible textile sensor consists of working and reference electrodes.

The in-textile wristband was fabricated on a single piece of commercial polyester [polyethylene terephthalate (PET)] cloth that processed high strength and resilience for universal civil and industrial applications. The PET cloth was metalized into conductive copper (Cu) cloth via the PAMD treatment. Modified double-sided photolithography was then applied for patterning the sensor electrodes and interconnects. The fabrication procedures are illustrated in Fig. 2A, including dip-coating of photoresist, soft baking for solvent removal, double-sided ultraviolet (UV) exposure for pattern coverage, pattern developing, hard baking for adhesion enhancement, wet etching for patterning, residues removal, and drying the cloth in the oven. An epoxy-silver glue was then applied to strengthen the adhesion between the rigid metal pins of electronic components and the Cu cloth. Unlike traditional photolithography for semiconductor technology, the metallic cloth was coated with photoresist by dip-coating rather than spin coating. Thus, conformal materials coating on the three-dimensional (3D) textiles network were formed by the interwoven warp and weft yarns. Figure. 2B shows that the as-fabricated PET cloth is ultra-flexible and can be twisted in versatile forms. The as-fabricated metallic pattern observed under a polarizing microscope showed sharp edges (Fig. 2C), indicating the high precision and controllability of the proposed in-textile metallization patterning method. Figure S2 (A to B) shows the PET and Cu cloth cross-sectional images under a scanning electron microscope (SEM). The images indicate that the metallic pattern is comparatively straight and has clean edges, demonstrating the high quality of the photolithographic circuit. Figure 2D shows that scalable patterns can also be realized with high fabrication efficiency and accuracy. It is worth mentioning that the photomask can be feasibly printed on transparent substrates by a commercial printer, which facilitates versatile circuit designs and large-scale pattern fabrication (fig. S3, A to B). Figure 2E shows the photograph of the as-fabricated in-textile K^+^ sensor. The integrated in-textile system was then encapsulated with insulating glue before being applied for in situ sweat analysis.

### Characterization of the monolithically integrated in-textile wristband

**Fig. 3.**
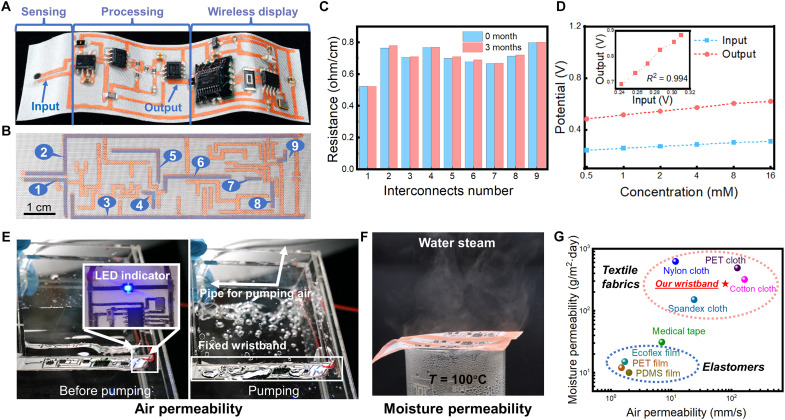
Characterization of the monolithically integrated in-textile wristband. (**A**) As-fabricated flexible in-textile sensing system. (**B**) Cu interconnects pattern on the textile. (**C**) Cu interconnect resistance over a shelf life of 3 months. (**D**) The OCP of the K^+^ sensor as input for the signal processing module and processed output verified with artificial sweat. The inset of (D) shows the voltage amplification accuracy. The input signal (blue) was tested by a voltmeter with artificial sweat in the range of 0.5 to 16 mM, and the same voltmeter tested the output signal (red) after being amplified and filtered by the signal processing module. (**E**) Photographs for air permeability test on bare PET cloth and waterproof sensing system with illuminated LED indicator. (**F**) Water vapor test of the as-fabricated biosensing wristband. (**G**) Air/moisture permeability of the as-fabricated biosensing wristband and the comparison with those of pure elastomers and textile fabrics. Elastomers: PET, polydimethylsiloxane (PDMS), and Ecoflex film. Textile fabrics: PET, nylon, cotton, and Spandex cloth.

The robustness of the monolithically integrated in-textile wristband was verified with comprehensive electrical and permeability characterizations. Figure 3A shows the digital photo of the monolithically integrated in-textile wristband. To enhance the electrical stability, the metallic interconnect pattern was covered with insulating Ecoflex. The encapsulation quality was verified by the water-immersed bending test, as shown in fig. S4. When immersing into the water, the interconnect covered with insulating Ecoflex maintained conductivity after 3000 bending cycles. The SEM photo also showed no delamination between Ecoflex and the interconnect, further offering evidence of its long-term stability after encapsulation. As shown in Fig. 3 (B and C), the resistance variations of each interconnect segment (marked with corresponding numbers) after 3 months were less than 1%, which indicates the long-term stability of the in-textile metallic interconnects as a hardware basis for the electrochemical sensor system. The OCP readouts of the sensor as the input (“Input” in Fig. 3A) for signal processing modules were linearly amplified (“Output” in Fig. 3A) to match with Bluetooth Low Energy (BLE) module parameters as shown in Fig. 3D. The voltage amplification had high goodness of fit of 0.994, which indicates the reliability of the custom-designed electronic module for biosignals processing. Attributed to the intrinsic fiber property and woven morphology, the in-textile system based on PET cloth delivered desirable air and moisture permeability. As shown in Fig. 3E and fig. S5, the as-fabricated in-textile integrated wristband was fixed onto an acrylic box, through which air was blown into the water by pumping. The air bubbles were observed passing through the as-fabricated textile (movie S1). The illuminated blue light-emitting diode (LED) mounted on the textile wristband verified the stable functionality and electrical conductivity of the integrated system underwater. In addition, the 3D porous structure of the textile effectively maintains the moisture breathability of the textile, as water vapor was observed to pass through the metallic patterned textile cover on top of a bottle of hot water (Fig. 3F). With delicate circuit interconnects patterning and packaging, the permeability of the wristband can be retained to the greatest extent. The in-textile wristband based on Cu-patterned PET cloth displayed excellent air permeability of 79 mm s^−1^ and moisture permeability of 270 g m^−2^ day^−1^, which are more than one order of magnitude higher than commercial medical tapes and elastomers that are commonly adopted in flexible electronics (Fig. 3G). In addition, the in-textile biosensing wristband shows competitive air/moisture permeability compared with commercial textile fabrics, which ensures the comfort of such wearable bioelectronics.

### Characterization of the electrochemical in-textile sensor

As a demonstration for sweat biomarkers analysis, a nanostructured electrochemical sensor was designed and fabricated on the Cu pattern PET cloth for K^+^ sensing (fig. S6). Sweat K^+^, as the basic cation in intracellular fluid, is an ideal biomarker for electrolyte imbalance. An undesirable decrease in the K^+^ concentration can be attributed to muscle and whole-body fatigue (*53*). The reduction of blood K^+^ concentration after exercise may be caused by the elimination of exercise stimulation and reflects as a significant decrease in sweat K^+^ concentration, which provides a theoretical basis for the study of K^+^ in sweat (*54*, *55*). The sensor was constructed on Cu-patterned cloths via electrochemical deposition methods. The bare Cu electrode conductors showed minimal resistance changes after 3000 cycles of bending test (fig. S7, A to B). To enable high sensitivity, the Cu electrode pattern was first decorated with nanodendritic gold (Au) as shown in figs. S8 and S9. The response signals were largely enhanced compared with the low sensitivity of 14 mV/decade for those without nanostructured layers (fig. S10A). PEDOT:PSS as the ion-electron transducer was then drop-casted on the electrode to improve long-term stability and suppress signal drift, compared with the sensors without PEDOT:PSS layer that delivers low sensitivity and a high voltage drift of 52 mV/hour (fig. S10B). Valinomycin is directly combined with other compounds to serve as the working electrode to achieve exclusive K^+^ recognition (*56*).

**Fig. 4.**
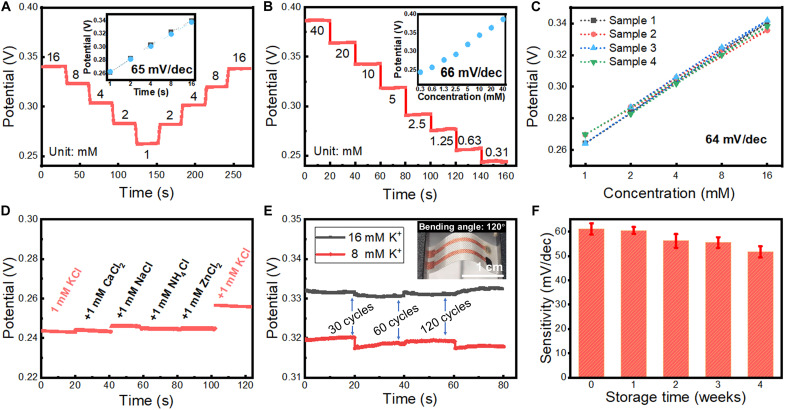
Performance evaluation of as-fabricated in-textile K^+^ sensor. (**A**) Repeatability of OCP responses of K^+^ sensor. The inset of (A) shows the corresponding calibration plots of the sensor. (**B**) The LOD of the in-textile sensor. The inset of (B) shows the corresponding sensitivity. (**C**) Reproducibility. (**D**) Selectivity. (**E**) Bending stability of the in-textile sensor. The inset of (E) shows the sensor after 120 cycles of bending. (**F**) Long-term stability. The error bars represent the RSD of the evaluated sensitivity from three samples.

The as-fabricated in-textile sensor delivered a sensitivity of 59 mV per decade and desirable repeatability (Fig. 4A and fig. S11). The K^+^ sensor showed a linear response in K^+^ concentration from 300 μM to 40 mM, which covers typical human sweat K^+^ levels within 1 to 18 mM (Fig. 4B) (*57*). Figure 4C shows that the sensor fabricated with the proposed process exhibited good reproducibility with a relative SD (RSD) of 2%. The selectivity of the sensor was investigated to ensure that the interference biomarkers (such as CaCl_2_, NaCl, NH_4_Cl, and ZnCl_2_) in sweat have limited effect on K^+^ monitoring (Fig. 4D and fig. S12). Moreover, the in-textile sensor maintains stable sensing output after different bending cycles of 30, 60, and 120 cycles, demonstrating the good flexibility and mechanical stability (Fig. 4E). In addition, the sensor showed a long shelf life with negligible sensitivity changes over 4 weeks with an RSD of 18% (Fig. 4F). The sensor signal drift was also carefully evaluated. Attributed to the large surface-to-area ratio of the nanostructured in-textile electrode and optimized functional material mass loading, the sensor drift was minimized to around 2.9 mV/hour at a concentration of 1 mM, as shown in fig. S13. The as-fabricated in-textile sensor shows competitive performance, which reveals that the as-fabricated electrochemical in-textile sensor with desirable sensitivity, selectivity, and stability can be applied for a reliable real-time sweat monitoring system. The above results show that the electrochemical sensors have competitive sensitivity, detection range, and limits of detection (LODs) compared with reported work, as shown in Table 1, indicating its prospect for efficient detection of the biomarkers in human body fluids.

**Table 1.** Various potentiometric sensors based on textiles in recent years.

Analyte	Fabric	Ion-electron Transducer	Sensing range (mM)	Sensitivity (mV)	Selectivity	Drift (mV/hours)	Bending duration (cycles)	Ref.
Na^+^	Silk	CNT	5–100	Na^+^: 51.8	Zn^+^, Ca^2+^, K^+^	–	60	(*24*)
pH, NH_4_^+^ Na^+^	PET fiber, steel thread	Conductive carbon ink	Na^+^: 1–1000; K^+^: 0.1–100; pH: 4–8	Na^+^: 52; NH_4_^+^: 60.8; pH: 62.3	K^+^, Na^+^, NH_4_^+^	2	–	(*26*)
K^+^, Na^+^	Polyurethane	CNT	0.1–100	K^+^: 56; Na^+^: 54	–	–	–	(*63*)
Na^+^	Carbon fibers	MWCNTs	1–100	Na^+^: 56	K^+^, Mg^2+^, Li^+^, Ca^2+^	1.3	–	(*66*)
pH	Cotton, silk and PET	Ag/AgCl nanoparticle	4–7	21	–	–	–	(*67*)
K^+^, Na^+^	Carbon fiber	PEDOT:PSS	K^+^: 0.1–100; Na^+^: 0.1–100	K^+^: 53.1; Na^+^: 57.9	NH_4_^+^, Ca^2+^, Mg^2+^	–	50	(*68*)
K^+^, Na^+^, Ca^2+^	CNT fiber	CNT	K^+^: 2–32; Na^+^: 10–160; Ca^2+^: 0.5–2.5	–	K^+^, Na^+^, Ca^2+^, NH_4_^+^	2.5	100	(*69*)
**K** ^**+**^	**PAMD-based cloth**	**PEDOT:PSS**	**0.3–40**	**66**	**pH, Ca** ^**2+**^ **, Na** ^**+**^ **, NH**_**4**_^**+**^ **, Zn** ^**2+**^	**2.8**	**120**	**This work**

### On-body application with the monolithically integrated in-textile wristband

**Fig. 5.**
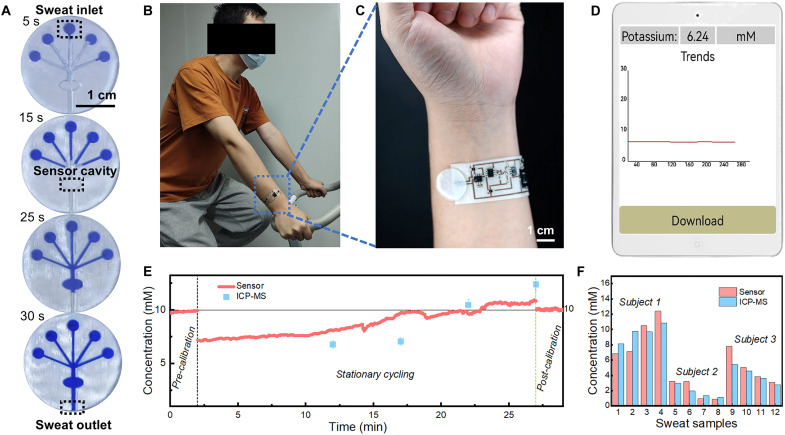
On-body application of the monolithically integrated in-textile wristband. (**A**) Verification for the microfluidic channel for artificial sweat collection in a flow velocity of 10 μl/min. (**B**) Photograph showing in situ sweat monitoring with the in-textile wristband during exercise. (**C**) Photograph of wristband mounted on the arm. (**D**) Custom-designed mobile application for real-time and continuous K^+^ sensing. (**E**) Representative real-time sweat potassium levels during stationary cycling. (**F**) Comparison of the extracted K^+^ concentrations by the sweat analysis wristband and ICP-MS.

To realize real-time and wireless sweat analysis, the as-fabricated in-textile sensor was integrated with a microfluidic module and custom-designed circuits in a wristband fashion. The microfluidic channels pattern was designed for sweat collection to facilitate efficient sweat collection and refreshing. Sweat was conveniently collected locally with the multi-inlet microchannel on a larger epidermal area (Fig. 5A). Wearable epidermal microfluidic devices can not only characterize the local sweat chemistry in freshly secreted sweat but also measure the average and instantaneous sweat loss for real-time analysis or subsequent studies. It has a variety of advantages, such as continuous sampling, real-time analysis, no contamination, and sweat evaporation (*58*). By using epidermal microfluidic channels as efficient sampling methods, wearable sweat devices enable autonomous and continuous sensing of vital electrolytes and metabolites in sweat with high sensitivity and reliability. Generally, a 1-cm^2^ skin area can cover 200 to 400 sweat glands on the arm based on estimation. On the basis of the sweat secretion rate (20 nl/min per gland) (*59*), the microfluidic module (3 cm^2^) is supposed to collect 12 μl of sweat per minute. Therefore, enough sweat could be drawn into the microchannel by hydraulic pressure from osmolality differences between sweat and plasma created by glands (*60*–*62*). The rate of sweat fluid flow through each “inlet” chamber is the same because of the symmetrical design, thus enabling continuous sweat analysis with improved reliability. Movie S2 also demonstrates that a fluid pump can pump artificial sweat into the channel and the artificial sweat can outflow the channel smoothly. The bottom layer, which is the double-sided adhesive layer in contact with the skin, was patterned with five sweat inlets to facilitate the sweat collection from a larger area and one sweat reservoir that covers the active sensing electrodes. The second layer (channel layer), in contact with the bottom layer, was prepared by polydimethylsiloxane (PDMS) on a reusable mold. This microfluidic module enabled a convenient and cost-effective method for contaminated module replacement (fig. S14, A to B).

As a proof of concept, the integrated wristband was placed on the arm of volunteers and the sweat K^+^ concentration can be continuously monitored and displayed on the custom-designed mobile application. (Fig. 5, B to D, and movie S3). The microfluidic channel cavity was filled after a warm-up of stationary cycling for around 20 min before initiating K^+^ sensing signal extraction. The sensor was calibrated with artificial sweat before and after the on-body test to ensure its sensing reliability. The real-time sweat K^+^ concentration tracking during physical exercise is shown in Fig. 5E. The on-body sweat tracking effectiveness can be further supported with more subject studies. The results are repeatable as shown in fig. S14 (C to D). Note that sensor contamination by the pollution from the skin surface, including dermal debris and sebum, is difficult to eliminate, although skin cleaning was performed. The decoded K^+^ concentrations extracted with the wristband were further validated by ICP-MS. In general, the perspiration fluctuation tendency from the sweat analysis patch is consistent with those from ICP-MS (Fig. 5F), indicating good reliability and great potential of the wearable sweat analysis patch for noninvasive human health monitoring.

## DISCUSSION

E-textiles have shown great promise for wearable healthcare applications mainly because of their unique properties of superior flexibility, intrinsic porosity, and air/moisture permeability, so as to enable long-term wearing comfort. While a variety of biosensors can be constructed on textiles, the entire sensing systems normally consist of rigid and airtight electronic components, and the integration of these multifunctional electronics mostly relies on external interconnects. This could introduce large impendence variation with mechanical interferences and thus limit the operational stability and wearing comfort of such textile-based systems (*63*–*65*).

In this work, an in-textile wristband with biosensors and supporting electronic modules monolithically integrated into a single piece of metallic cloth was demonstrated as a highly stable platform with wearable comfort for wireless epidermal biomarker analysis. A facile and versatile approach using PAMD and double-sided photolithography was exploited to achieve the monolithic fabrication of electrodes and circuit interconnects on textiles while maintaining the inherent porous textile structures. Such in-textile conductive patterns deliver excellent electrical and mechanical stability with proper insulating glue encapsulation, showing negligible electronic conductivity variation during bending tests and 3 months of shelf life. Moreover, the metallic patterned textiles have excellent air/moisture permeability and can even perform electronic functionality underwater. As a proof of concept, the conductive electrodes were functionalized into selective K^+^ sensors, and a superior sensitivity of 66 mV/decade with reproducibility of 2% RSD was achieved. Moreover, with proper electronics integration and by using the epidermal microfluidic module as an efficient epidermal sweat sampling method, the as-fabricated wearable wristbands can continuously monitor the K^+^ level in epidermal sweat. The reliability of on-body sweat analysis using the wristband was validated by comparing the extracted biosensing results with standard analytical tools. The strategy developed in this work to realize such a monolithically integrated in-textile biosensing system can also be leveraged into various custom-designed systems based on amperometry, cyclic voltammetry, and impendence sensing with wireless protocols. It would no doubt inspire further advances in multifunctional E-textiles with desired stability and wearing comfort for fitness monitoring, personalized daily health care, and the Internet of Medical Things.

## MATERIALS AND METHODS

### Materials

Sodium hydroxide (NaOH), ethyl alcohol, acetic acid, 3-(trimethoxysilyl) propyl methacrylate (KH570), methacrylatoethyl trimethyl ammonium chloride (METAC), potassium peroxodisulfate (K_2_S_2_O_8_), ammonium tetrachloropalladate (II), potassium sodium tartrate, copper sulfate pentahydrate, formaldehyde solution, and chloroauric acid (HAuCl_4_) were purchased from Sigma-Aldrich. Hydrochloric acid (HCl), bis(2-ethylehexyl) sebacate (DOS), sodium tetrakis [3,5-bis(trifluoromethyl)phenyl] borate (Na-TFPB), high–molecular weight polyvinyl chloride (PVC), valinomycin (potassium ionophore), cyclohexanone, and tetrahydrofuran were purchased from J&K Scientific. PEDOT:PSS (PH1000), potassium ferricyanide (III), chloroauric acid, silver paste, PVB resin BUTVAR B-98, sodium chloride (NaCl), iron (III) chloride (FeCl_3_), and potassium chloride (KCl) were purchased from Aladdin Biochemical Technology. All other chemicals were commercially available and used without further purification. All solutions were prepared using deionized (DI) water (16 megohm·cm) produced from a Millipore water purification system.

### Fabrication of metallic cloth

The PAMD process was conducted on commercial PET cloth. PET cloth was cleaned with ethanol and then immersed in a 2 M NaOH solution for 1 hour at 80°C to generate hydroxyl groups on the surface. Afterward, PET cloth was cleaned with DI water and dried by air dry oven. Subsequently, PET cloth was then immersed in a 4% (v/v) KH570 solution for 1 hour to allow the reaction of silane with hydroxyl groups on the surface. The solvent comprised 95% ethanol, 1% acetic acid, and 4% DI water. The KH570-modified PET cloth was then immersed in a 20% (v/v) METAC aqueous solution for 1 hour at 80°C to carry out in situ free-radical copolymerization on the substrate surface using K_2_S_2_O_8_ as an initiator. As a result, METAC-modified PET substrates were obtained. Then, the cloth was soaked into a 4 × 10^−3^ M (NH_4_)_2_PdCl_4_ aqueous solution. During soaking, the [PdCl_4_]_2_− catalytic ions were immobilized onto the quaternary ammonium groups of poly-METAC polymer chains through ion exchange. Afterward, DI water was infused to rinse the physically adsorbed catalyst. Cu-PET cloth was fabricated by applying a typical Cu electroless plating solution by the Cu plating bath consisting of a 1:1 mixture of freshly prepared solutions A and B. Solution A contained NaOH (12 g/liter), CuSO_4_·5H_2_O (13 g/liter), and potassium sodium tartrate (29 g/liter) in DI water. Solution B was a formaldehyde (45 ml/liter) aqueous solution. After electroless deposition, all samples were rinsed with DI water and dried by compressed air.

### Fabrication of metallic electrodes and interconnects on textile

The double-sided lithography approach was adopted for fabricating the electrode and circuit. The metallic cloth was first covered with photoresist (NR9-1500p, Futurrex, USA) by dip-coating. Next, the photoresist-covered textile was heated in an oven at 120°C for 5 min to solidify the photoresist. Then, the sample was exposed to a double-sided UV light for 1 min with two same masks, followed by heating in an oven at 120°C for 3 min to promote the photoresist reaction. The photoresist in the nonpatterned area of the sample was removed by a developer solution (RD6, Futurrex, USA). Then, the metal in the nonpatterned area was etched in 1 M FeCl_3_. Last, the photoresist on the metal pattern was washed away in acetone. The metallic patterned textiles were then rinsed with alcohol and DI water before drying in the ambient for further process.

### Fabrication of sensor electrode

The K^+^-selective membrane cocktail was composed of valinomycin (2% w/w), sodium tetraphenylboron (Na-TPB) (0.5% w/w), PVC (32.5% w/w), and DOS (65% w/w). One hundred milligrams of the membrane cocktail was dissolved in 660 μl of tetrahydrofuran. The ion-selective solutions were sealed and stored at 4°C. The electrolyte for Au dendritic nanostructure growth was a mixture of 50 mM HAuCl_4_ and 50 mM HCl. The deposition was conducted by applying a periodic voltage wave with an amplitude of −2 V, frequency of 50 Hz, and duty cycle of 50% in the electrolyte for 9000 cycles. PEDOT:PSS was adopted as the ion-electron transducer and deposited onto the working electrode. PEDOT deposition was realized by drop-casting 3 μl of PEDOT:PSS (PH1000) on the electrode. Ion-selective membranes were then prepared by drop-casting 6 μl of the K^+^-selective membrane cocktail onto the PEDOT:PSS/Au electrode. The reference electrode for the K^+^ sensor was modified by casting 10 μl of reference solution onto the Ag/AgCl electrode. The solution for the PVB reference electrode was prepared by dissolving 79.1 mg of PVB and 50 mg of NaCl into 1 ml methanol. The modified electrode was left to dry overnight. The ion-selective sensor was stabilized in the solution containing 1 mM KCl for 4 hours before measurements to obtain the best performance for long-term continuous measurements. The conditioning process was important to enhance stability and minimize potential drift.

### Assembling of the monolithically integrated in-textile wristband

The signal processing module mainly used three LMV358 chips as a low-power operational amplifier with a rail-to-rail output swing, a STC8H3K64S2 microcontroller, and a Bluetooth chip (CH9141K). The extracted sensor OCP was first amplified to millivolt levels before noise suppression by the low-pass filter. The microcontroller for signal processing can be programmed by the software KELI. A 12-bit analog-to-digital converter module within the microcontroller converted the analog signal into a digital signal. Then, the microcontroller processed and transmitted the data to the phone via the Bluetooth chip. A small-capacity (40 mA·hour) lithium-ion battery can directly power the whole system at a nominal voltage of 3.7 V.

### On-body sweat analysis

All experiments were performed according to the university guidelines (The Ethics Guidelines for Research Involving Human Subjects or Human Tissue from Southern University of Science and Technology, SUSTech Institutional Review Board, 20200037). On-body evaluation of analysis in sweat was performed on healthy volunteers aged between 24 and 26 years. For real-time K^+^ monitoring, the wristband was connected to the smartphone application via Bluetooth. Volunteers were first asked to wear the wristband and cycled for around 20 min as a warm-up process. After the sweat fills the cavity, the volunteers perform a half-hour stationary cycling. Meanwhile, the application connection was started to start data recording, and decoded sweat analysis results were displayed on the cellphone. Sweat was simultaneously collected every 5 min to compare sensor data with measurements from ICP-MS.

### Ex situ evaluation of the sweat samples

Ex situ sweat sensing was also conducted with exercise-induced sweat collected from the subjects’ arms near the epidermal areas where the sensing patch was worn. Sweat samples were collected with microtubes every 5 min (except for the first 10 min for warm-up). The areas were wiped and cleaned with gauze after sample collection. The sweat samples were temporarily stored in the refrigerator at −4°C. For ICP validation, 4 μl of collected sweat sample was mixed with 3 ml of standard buffer solution for each test.

### Fabrication of the microfluidic channel

The template of the microfluidic channel was printed using a 3D printer (Adventurer3) at a speed of 30 mm/s. PDMS was homogeneously mixed at a 10:1 weight ratio of base to curing agent and solidified at 60°C in a high-temperature oven for 3 hours. For pattern design, the diameter for five cylindrical sweat inlets was 3 mm, and the channel size for perspiration flow was 0.3 mm by 0.3 mm by 10 mm. The size of the elliptical sensor cavity was 3 mm by 6 mm, which could fit the sensor electrodes exactly. The cured PDMS with microfluidic pattern was then peeled from the template and attached onto the double-sided medical tape to form microfluidic channels. On the other side of the double-sided medical tape, the sensor part of the integrated band was attached beneath the microfluidic module, and the integrated patch can be adhered onto skin.

### Characterization

The microstructures of samples were observed by SEM (Hitachi TM3000) and polarizing microscope (Nikon). Electrodeposition and sensor performance were performed by two types of electrochemical workstations (CHI660e and IviumSoft 4). To investigate the stability of the sensors, the performance of three samples was recorded each week over 4 weeks, and at other times, the samples were stored at room temperature (25°C) to preserve the activity of the ionophore on the sensor. In addition, the K^+^ concentrations in the collected sweat samples were also validated using ICP-MS (Thermo Fisher Scientific Q Exactive). The air permeability value (in mm s^−1^) of all the samples was tested according to ASTM D737-08 using a MO21S air permeability tester (SDL America Inc.). Moisture permeability tests for the samples were performed using the cup method according to the textile standard E96/E96M-13. The moisture transmission rate (g m^−2^ day^−1^) was determined by measuring the weight loss of the water vapor in a cup with its opening firmly covered by the tested specimen. Both air resistance and moisture permeability tests were performed at around 22°C and 63% relative humidity (testing duration of 1 week). The mechanical properties of the sensor and Cu patterned cloth were tested using an Instron 5599 universal testing system. The electrical properties of the samples under different stretching states were investigated by a Keithley 2400 SourceMeter coupled with a computer-controlled stretching motor.
