# Supplementary material for: A monolithically integrated in-textile wristband for wireless epidermal biosensing
Source: Sci Adv. 2023 Nov 10;9(45):eadj2763. doi: 10.1126/sciadv.adj2763 (PMC10637736; doi:10.1126/sciadv.adj2763)
Supplement: Supplementary file 1 — Figs. S1 to S14 Legends for movies S1 to S3 Legend for data S1 [file sciadv.adj2763_sm.pdf]

Science Advances
AAAS

Supplementary Materials for

A monolithically integrated in-textile wristband for wireless epidermal biosensing

Xiaohao Ma *et al.*

Corresponding author: Yuanjing Lin, linyj2020@sustech.edu.cn; Zijian Zheng, zijian.zheng@polyu.edu.hk

Sci. Adv. **9**, eadj2763 (2023)
DOI: 10.1126/sciadv.adj2763

The PDF file includes:

Figs. S1 to S14
Legends for movies S1 to S3
Legend for data S1

Other Supplementary Material for this manuscript includes the following:

Movies S1 to S3
Data S1

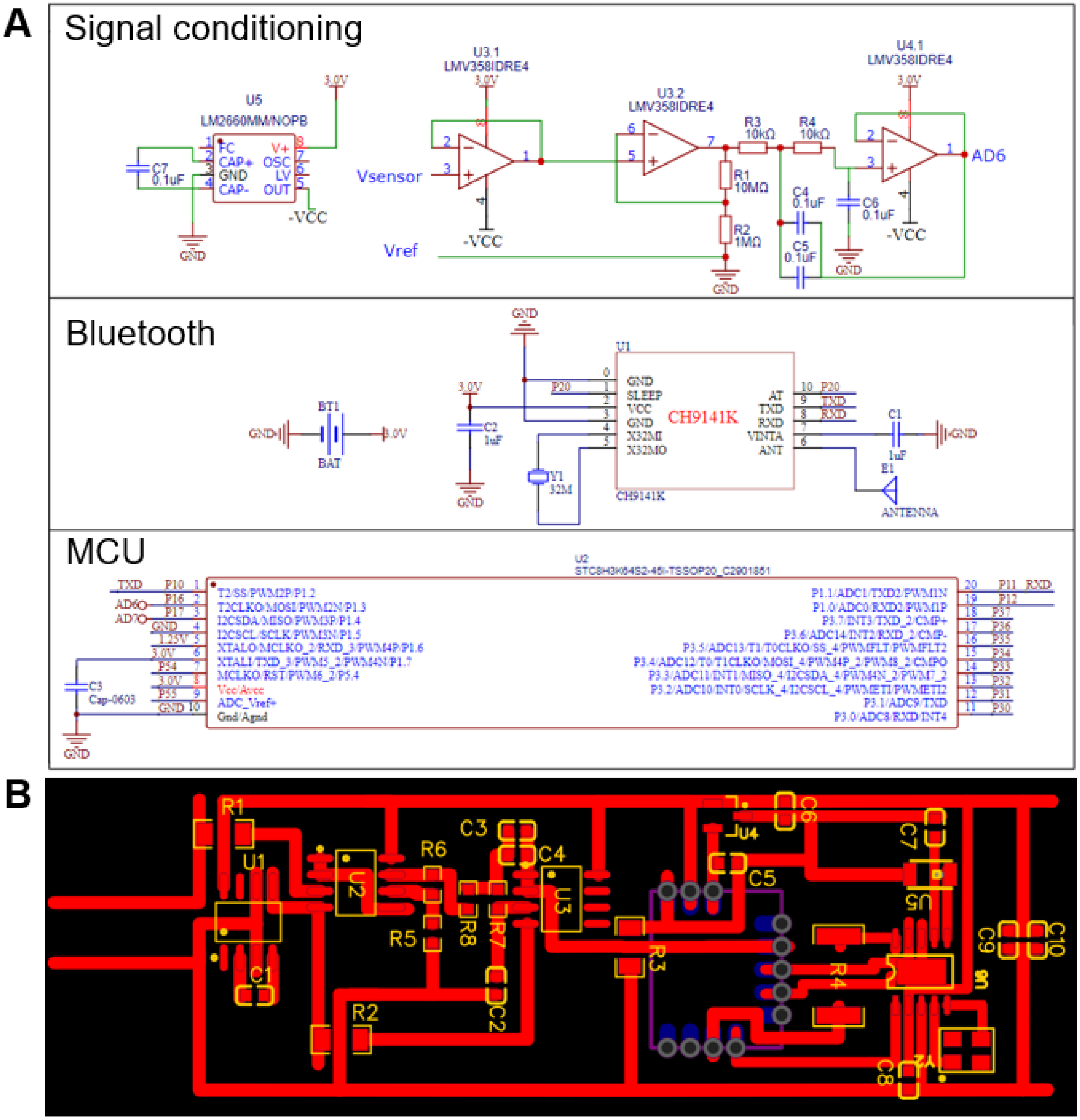

Fig. S1. Schematic and circuit design of the in-textile wristband. (**A**) Schematic diagram of signal-conditioning (voltage follower, amplifier, 2nd order low-pass filter) and wireless circuit. (Bluetooth, ADC part). (**B**) One-layer circuit design based on Cu cloth (3×8 cm).

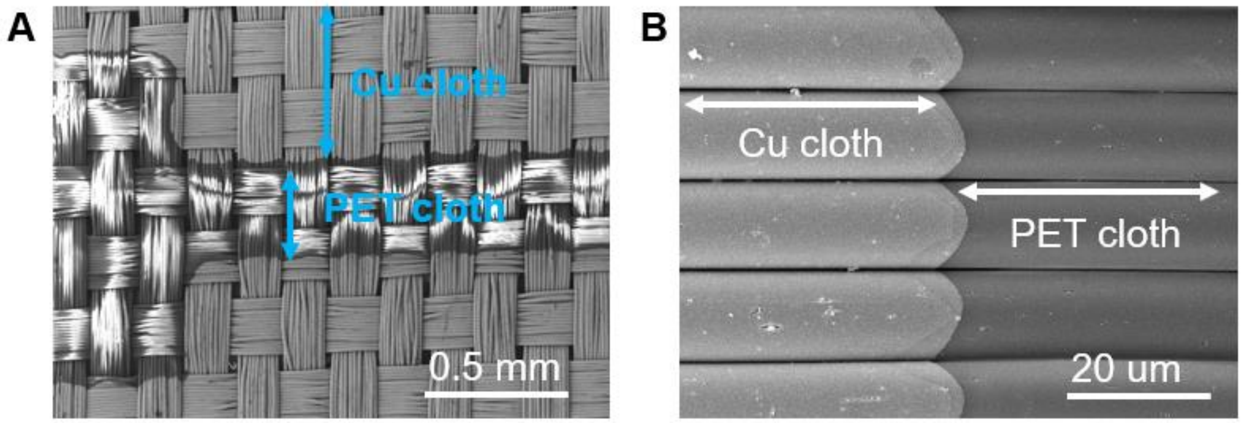

Fig. S2. Cross sections for (**A**) sensor electrode and (**B**) etched Cu cloth.

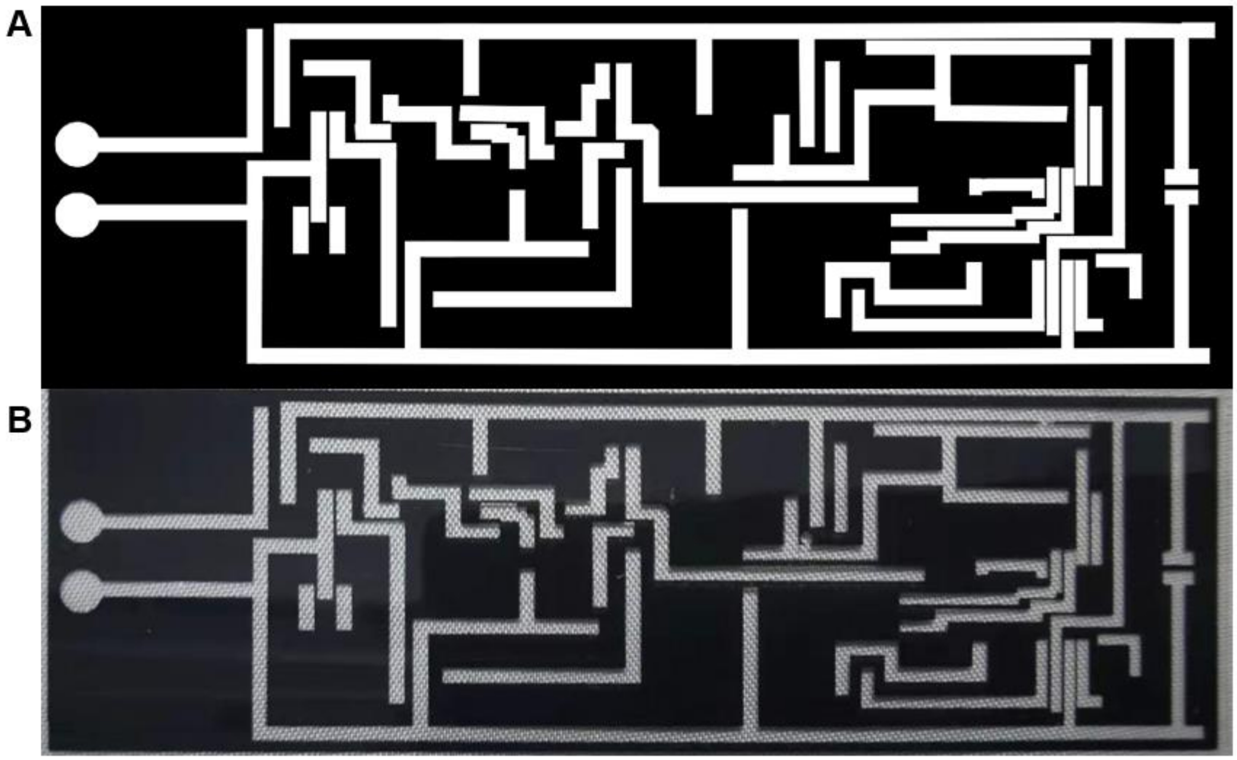

Fig. S3. Photolithography mask design and pattern. (**A**) One layer circuit connection design (line width=1 mm). (**B**) Mask printed onto transparent A4 thin film.

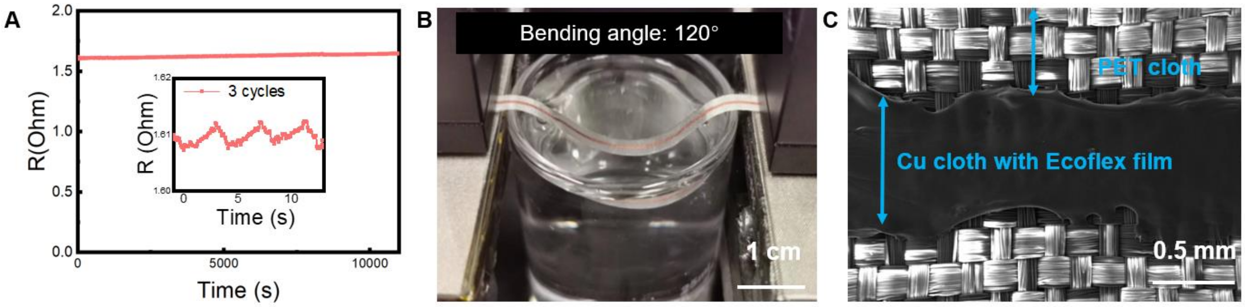

Fig. S4. Bending characterization of the interconnect glued with Ecoflex. (**A**) Resistance changes over 3000 bending cycles. (**B**) The setup for bending and insulation test. (**C**) SEM photo after bending 3000 cycles.

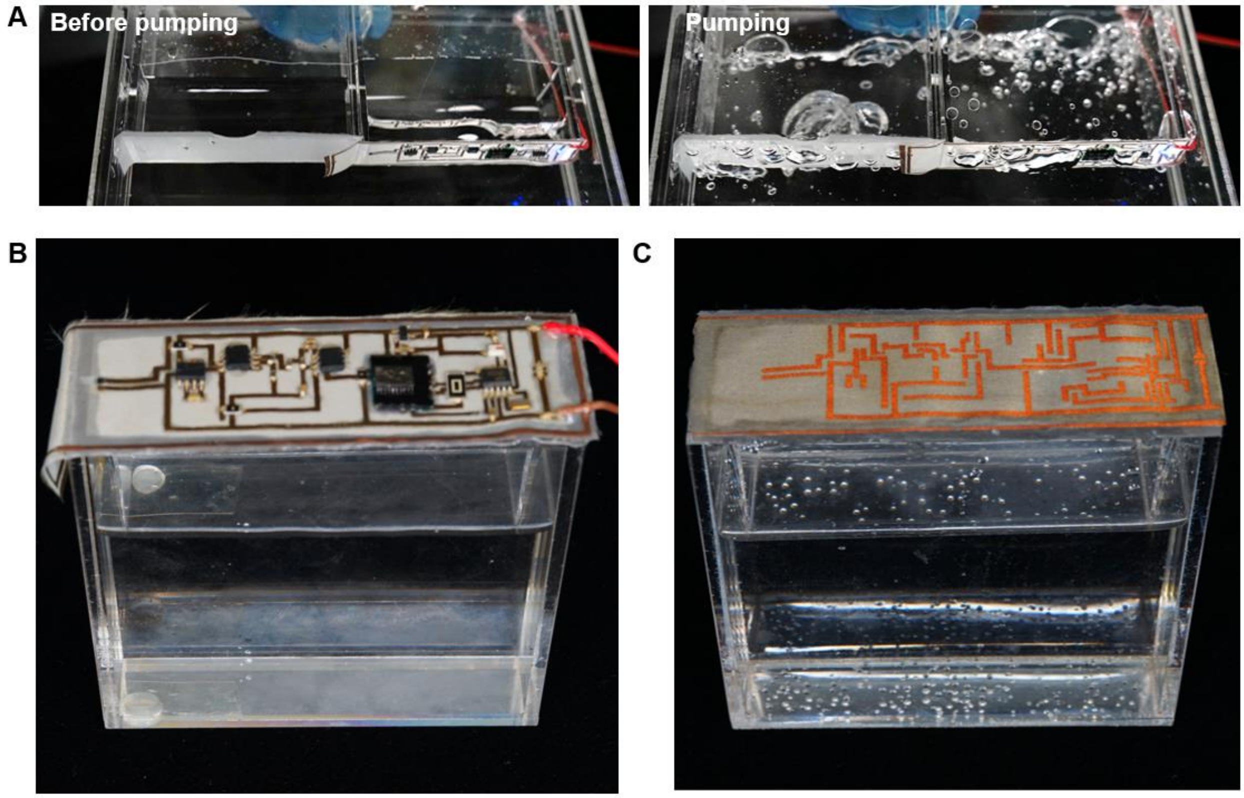

Fig. S5. Air and moisture permeability characterization. (**A**) Air permeability test for the textile wristband. The setup for air and moisture permeability test for (**B**) integrated in-textile electronic system and (**C**) metal patterned in-textile interconnects.

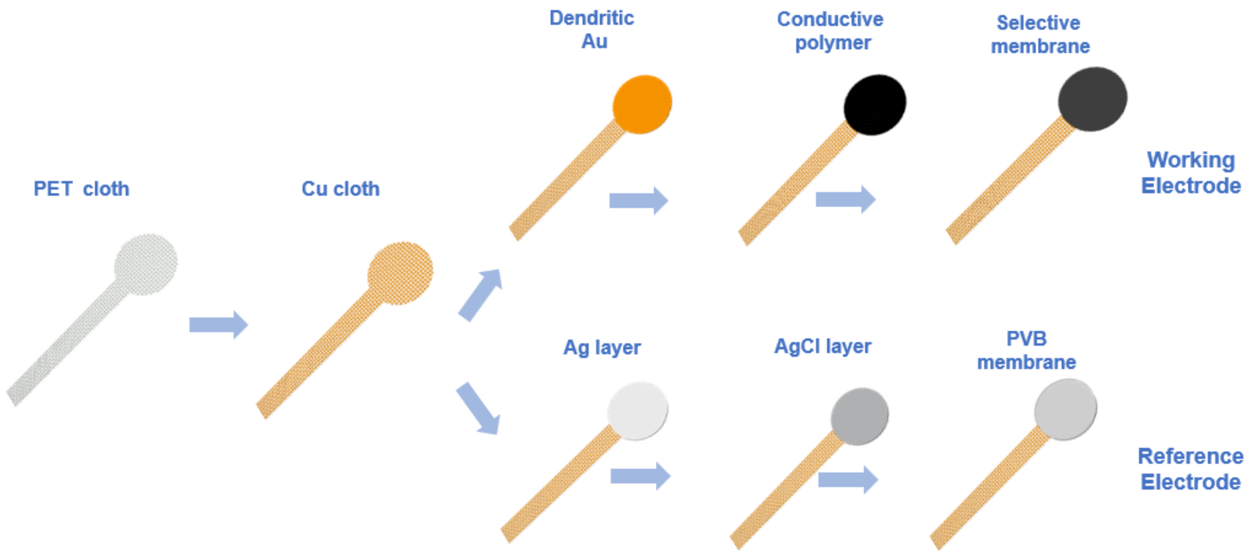

Fig. S6. Fabrication of working electrode and reference electrode for the in-textile sensor.

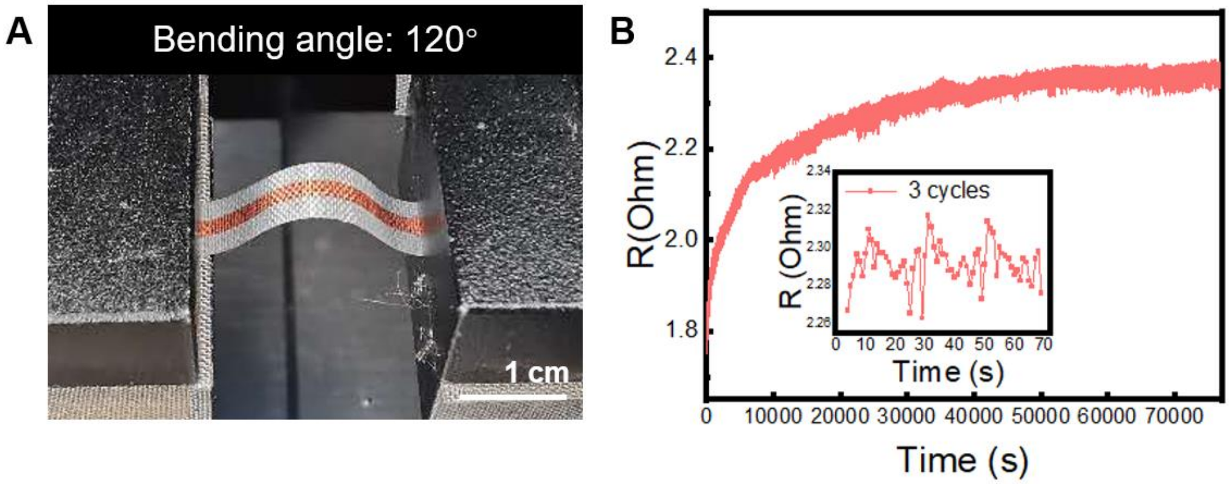

Fig. S7. The bending test of Cu cloth. (**A**) Photograph for bending Cu cloth. (**B**) Resistance changes for Cu cloth over 3000 bending cycles.

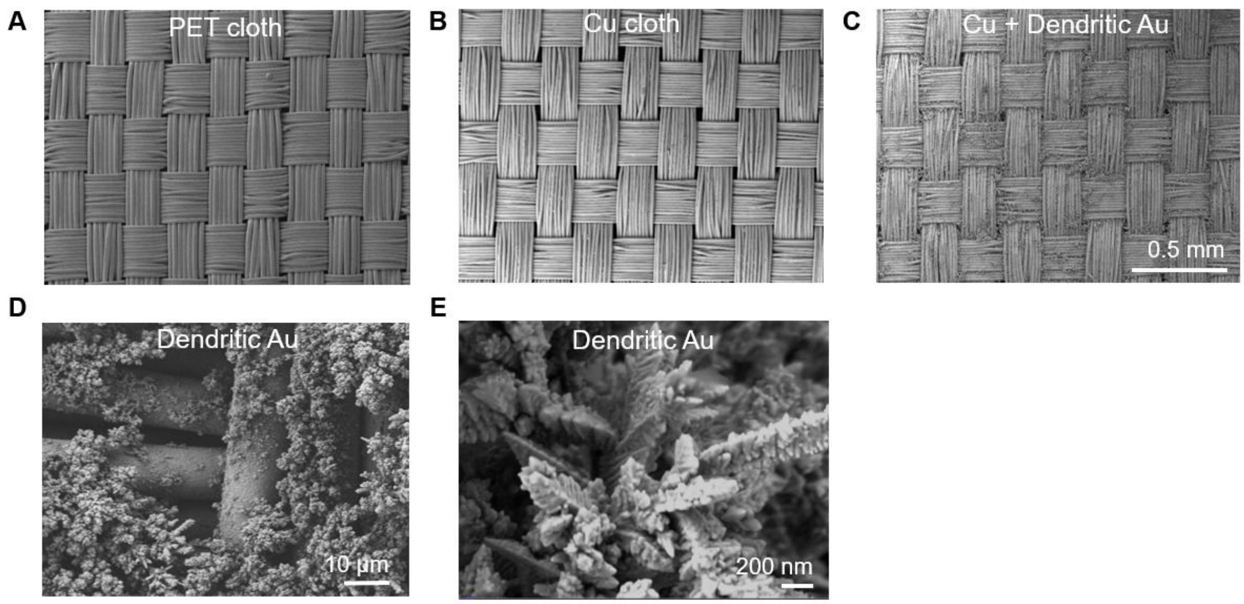

Fig. S8. SEM characterization of (**A**) PET cloth. (**B**) Cu cloth. (**C**) Dendritic Au onto Cu cloth. (**D**)&(**E**) Dendritic Au grows from fabric fibers.

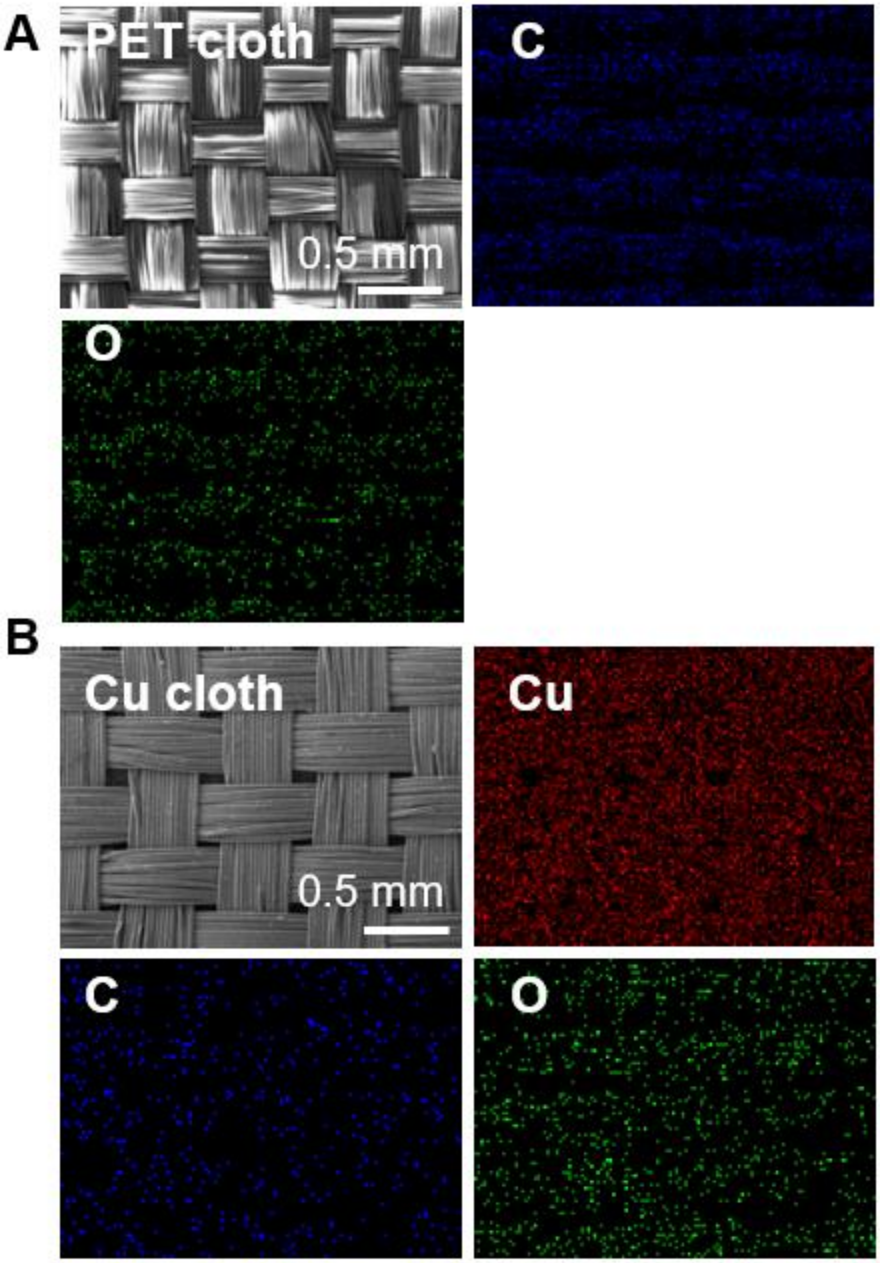

Fig. S9. Energy Dispersive X-ray (EDX) spectroscopy characterization of (**A**) PET cloth. (**B**) Cu cloth.

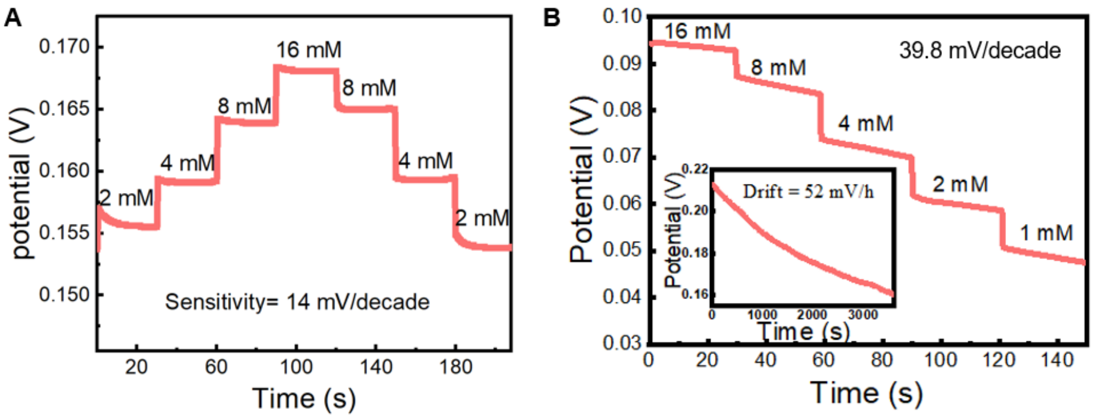

Fig. S10. Performance Sensitivity and drift of sensors (**A**) Without dendritic Au layer. (**B**) Without PEDOT: PSS.

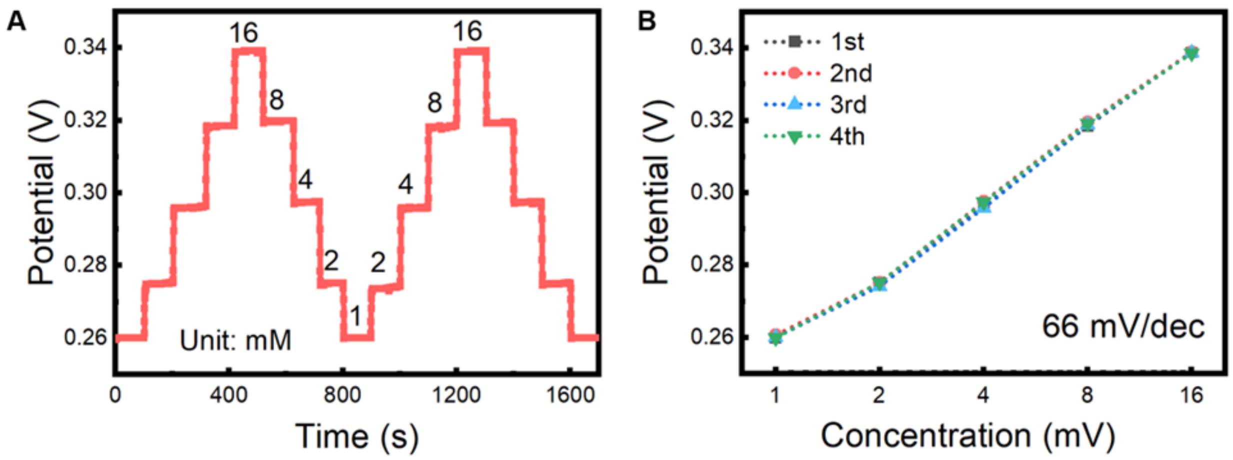

Fig. S11. The cycling stability of a K^+ sensor. (**A**) Potentiometric responses in four cycles and (**B**) the calibrated sensitivities.

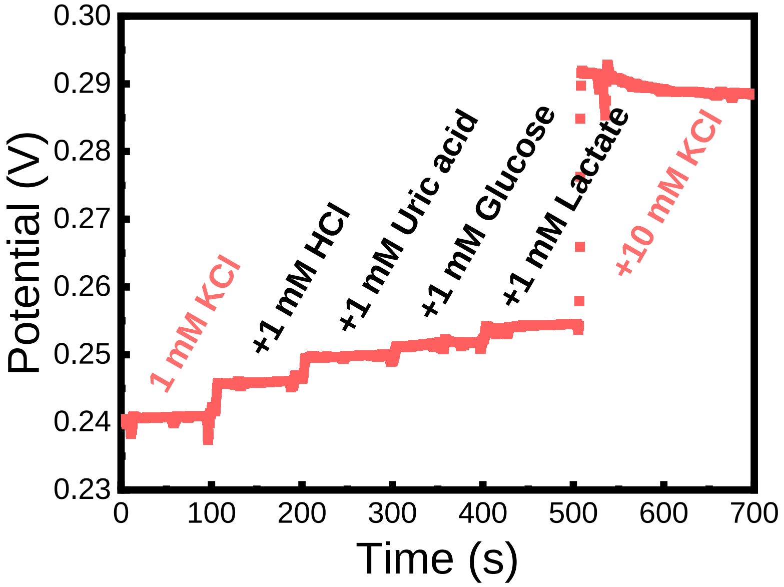

Fig. S12. Selectivity for pH changes and presence of molecular metabolites.

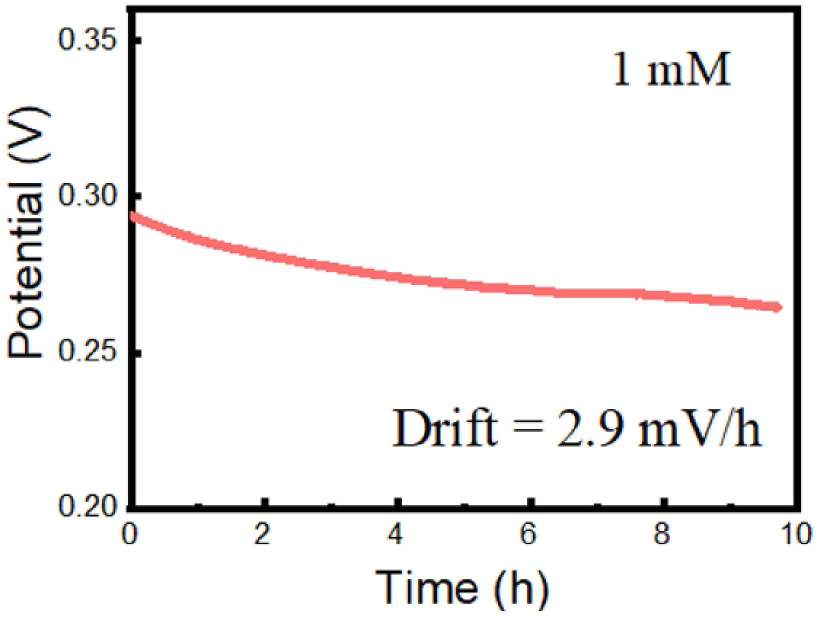

Fig. S13. Sensor performance for voltage drift at 1 mM.

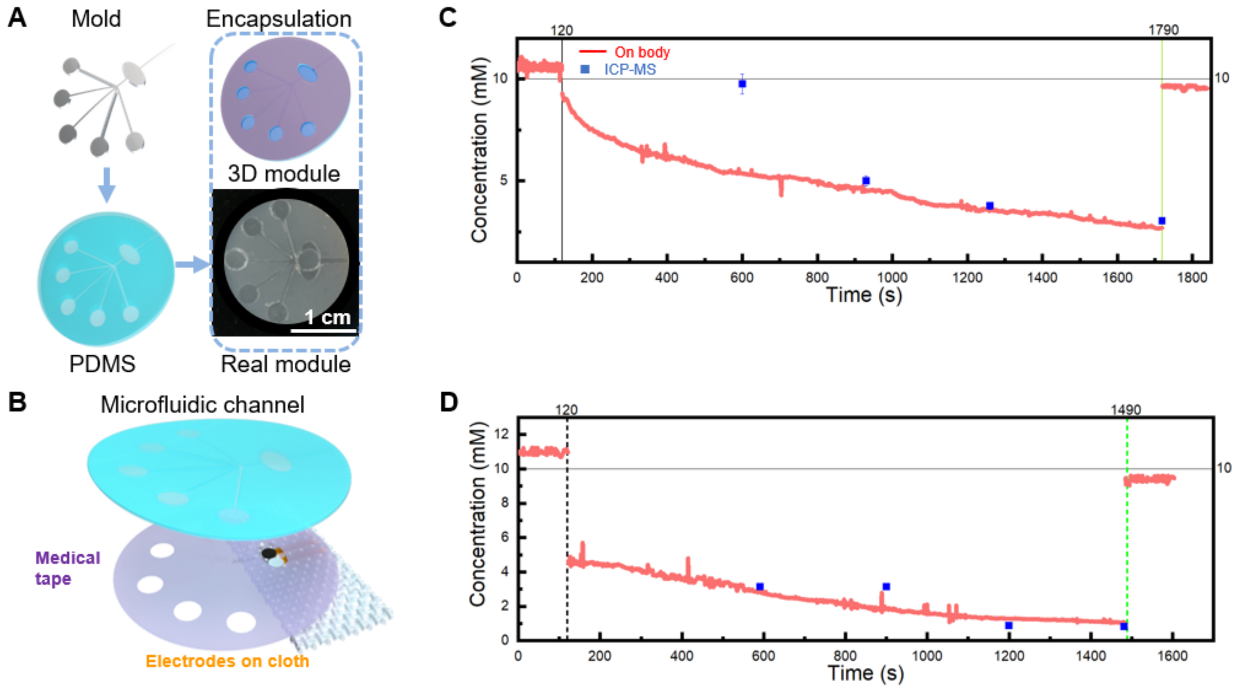

Fig. S14. Repeatable sweat analysis based on microfluidic module. (**A**) Fabrication of microfluidic channels. (**B**) Illustration of the function of microfluidic channels. (**C**)&(**D**) On-body real-time perspiration analysis on a subject's wrist during stationary cycling.

Movie S1.
The in-textile system based on PET cloth with desirable air permeability and waterproofness.

Movie S2.
Verification of the microfluidic module when pumping the artificial sweat through the channel.

Movie S3.
On-body real-time perspiration analysis on a subject’s wrist.

Data S1.
The main code for signal processing and wireless display. (Programmed by the software Keli)
